# Tuning the Nature of N-Based Groups From N-Containing Reduced Graphene Oxide: Enhanced Thermal Stability Using Post-Synthesis Treatments

**DOI:** 10.3390/nano10081451

**Published:** 2020-07-24

**Authors:** Stefania Sandoval, Gerard Tobias

**Affiliations:** Institut de Ciència de Materials de Barcelona (ICMAB-CSIC), Campus de la UAB, 08193 Bellaterra (Barcelona), Spain

**Keywords:** graphene, thermal stability, doping, graphene, graphene oxide, N-oxides

## Abstract

The synthesis of N-containing graphene derivatives by functionalization and doping of graphene oxide (GO) has been widely reported as an alternative to tune both their chemical and physical properties. These materials are of interest for a wide range of applications, including biomedicine, sensors, energy, and catalysis, to name some. Understanding the role of the nature, reactivity, concentration, and distribution of the N-based species, would pave the way towards the design of synthetic routes to obtain improved materials for specific applications. The N-groups can be present either as aliphatic fractions (amides and amines) or becoming part of the planar conjugated lattice (N-doping). Here, we have modified the distribution of N-based moieties present in N-containing RGO samples (prepared by ammonolysis of GO) and evaluated the role of the concentration and nature of the species in the thermal stability of the materials once thermally annealed (500–1050 °C) under inert environments. After these post-synthesis treatments, samples underwent marked structural modifications that include the elimination and/or transformation of N-containing fractions, which might account for the observed enhanced thermal stability. It is remarkable the formation of pyridinic N-oxide species, which role in the properties of N-containing graphene derivatives has been barely reported. The presence of this fraction is found to confer an enhanced thermal stability to the material.

## 1. Introduction

Graphene is the basic structural parent of carbon nanomaterials, which include 3D graphite, 1D carbon nanotubes, and 0D buckyballs [1]. It was theoretically described by P. R. Wallace in 1947 [2] but its isolation and experimental properties were not described until 2004 by Novoselov et al. [3]; becoming one of the most studied structures in materials science. Graphene consists of a single-atomic layer, constituted by hexagons of sp^2^ hybridized carbon atoms forming a honeycomb lattice (trigonal planar structure). This structure is responsible of its remarkable properties [4]. Graphene exhibits large surface area (up to 2600 m^2^·g^−1^), high thermal conductivity (ca. 5000 W·m·K^−1^), fast charged carrier mobility (ca. 200,000 cm^2^·V^−1^·s^−1^), strong Young’s modulus (ca. 1 TPa) and extraordinary robustness [1,5]. Graphene finds application in different fields, such as batteries, sensors, and biomedicine [6]. Its physical and chemical properties are associated with the unique energy-band structure and how the electrons behave in the lattice (Dirac fermions). Therefore, it is possible to modify these properties by tailoring its electronic structure. This might be achieved by chemical modifications of the graphene lattice [7,8,9,10], as well as by the decoration of its basal plane with inorganic nanoparticles leading to nanocomposites with applications in MRI [11], antimicrobial/antibacterial agents [12,13], cathode material for batteries [14] and biosensors [15], to name a few.

Oxygen-based moieties present in graphene oxide (GO) are susceptible of interacting with a myriad of species under appropriate conditions. GO is usually employed as a precursor for the preparation of graphene derivatives, as it is the case of N-containing graphene. The latter can be prepared by both in situ treatments, which include CVD [16] and arc discharge reactions [17], or via post synthesis approaches [18], such as plasma [19], ammonolysis [20,21,22], hydrothermal [23,24], laser [25] or aerosol-based processes [26]. All of them have been reported as successful strategies to introduce N within the GO structure [27]. Ammonolysis of GO is, in particular, an easy and versatile tool for the synthesis of N-containing RGO (NRGO). By controlling the temperature (T) during the ammonia treatment, not only the level of reduction, but also the nature of the N-based functionalities can be easily tuned. Aliphatic moieties, namely amines and amides can be selectively obtained when using low temperatures of treatment (room temperature or 100 °C). The formation of more stable species (N-doping) requires the use of higher temperatures. When increasing T, a variety of materials can be obtained. In samples prepared in the range of 220–400 °C, both aliphatic and structural N coexist, while using T ≥ 500 °C leads to the formation of N-doped RGO [21,22].

The synthetic approach plays a determinant role in the characteristics of the final product. Reactions of nitrogen sources with sp^3^ atoms (usually from the edges) lead to the formation of N-functionalized samples (introducing aliphatic moieties; amines and amides) while preserving the sp^2^ conjugated system. When the defect sites are located in the basal plane, nitrogen can be introduced into the graphene lattice (N-doping), forming three main bonding configurations: pyridinic, pyrrolic, and quaternary N (or graphitic N) [28]. Each of these N-groups confers differentiated electronic and transport properties to the final product. In the case of post synthesis treatments, the introduced N-containing moieties depend on the initial distribution and nature of aliphatic defects present in the GO precursor. Although less frequent, one additional N-oxide pyridinic configuration (C_5_H_5_N^+^–O^−^) can be formed during the syntheses [27]. This heterocycle fraction consists in a six-membered ring formed by 5 C atoms and 1 N, which is also linked to an oxygen. Despite it having been previously reported, its presence and effects within the graphitic honeycomb lattice has been barely analyzed. The ambivalent character of this group, which configuration induces a dipole moment, is remarkable. It can react with electrophilic or nucleophilic reagents, thus becoming a versatile fraction susceptible of being derivatized.

Reports on N-doped RGO have shown that the material turns more stable against thermal oxidation [29], which extends its potential application for fuel cell operation and other fields where high temperatures are required [8,30,31]. Thus, N-doped RGO has been proposed as a reinforcement for systems exposed at elevated temperatures and friction [32] since it can be integrated into 3D structures [33]. Other applications include supercapacitors [34], sensors [35], electrocatalysis [36,37], solar cells [38] and as a magnetic material [39,40].

The stability and concentration of the N-containing moieties might play a role in the physical and chemical properties of these materials. Herein we report on the thermal stability against oxidation and the variation in the distribution of different N-based fractions present in N-containing RGO samples that have undergone a thermal annealing under N_2_, Ar, or Ar/H_2_.

## 2. Materials and Methods

### 2.1. Reagents

Graphite flakes (< 150 µm, value provided by the supplier, Sigma-Aldrich, MO, USA), NaNO_3_ (> 99%, Sigma-Aldrich, Steinheim, Germany), KMnO_4_ (> 99%, MO, USA), 2-propanol (anhydrous, 99.5%, Steinheim, Germany) and H_2_O_2_ (30%, Panreac química, SLU, Darmstadt, Germany) were used as received. NH_3_ gas (99.99%) and N_2_ (99.9997%) were provided by Carburos Metálicos (Spain) and Ar (99.9999%) by Air Liquide (Spain).

### 2.2. Synthesis of N-Contanining Reduced Graphene Oxide (RGO) Samples

One-hundred milligrams (100 mg) of graphene oxide (GO), prepared by a modified Hummers’ method [29], were spread into a sintered Al_2_O_3_ boat and placed into a silica furnace tube. Afterwards, the sample was annealed during 1 h at temperatures ranging between 220 °C and 800 °C in the presence of pure ammonia gas, flowing at 300 mL·min^−1^ [21].

### 2.3. High Temperature Treatments of N-Containing RGO

NH_3_-treated GO samples were dried and ground using an agate mortar and pestle, spread into a sintered alumina boat (Al_2_O_3_, ALSINT 99.7%, Keratec) and placed into a silica furnace tube. The sample was located in the center of a tubular furnace on top of the thermocouple. The system was initially purged with the corresponding gas for 2 h at a flow rate of 150 mL·min^−1^ in order to eliminate the oxygen present. The samples were annealed during 2 h at temperatures ranged between 500 °C and 1050 °C under different atmospheres (Ar, N_2_, and Ar/H_2_).

### 2.4. Characterization

Elemental analyses were performed on a Thermo Scientific™ FLASH 2000 Series CHNS Analyzer (Waltham, MA, USA) using a Mettler Toledo MX5 microbalance.

The morphology of the sheets was studied recording SEM images using a Quanta FEI 200 ESEM FEG microscope (Hillsboro, OR, USA), operating at 10.0 kV. Samples were prepared dispersing the sample in hexane and subsequently placing it dropwise onto a lacey carbon support grid. Thermogravimetric analyses were performed on a Netzsch instrument, model STA 449 F1 Jupiter^®^ (Selb, Germany), under flowing air at a heating rate of 10 °C·min^−1^.

X-ray photoelectron spectra (XPS) were recorded in a Kratos AXIS ultra DLD spectrometer (Manchester, UK) using monochromatic Al Kα. All samples were located in the preparation chamber as received and on the same Cu substrate to maintain the analysis conditions invariable. High-resolution spectra of C1s, O1s, and N1s regions were registered.

Fourier transform infrared (FT-IR) spectra were recorded in transmission mode on a JASCO FT-IR 4700 spectrometer (Tokyo, Japan), in the range of 2000 cm^−1^ to 500 cm^−1^. The samples were prepared by dropping dispersions of the materials (previously prepared by sonication of the powder in 2-propanol, ca. 10 mg∙mL^−1^) onto preheated ZnSe disks (2 mm thickness, 80 °C).

### 2.5. Nomenclature of the Samples

For ease of discussion, we will refer to the GO samples treated under ammonia (nitrogen-containing reduced graphene oxide) as NRGO. The name of the sample will be followed by a number that corresponds to the temperature that has been used for the ammonolysis treatment. Finally, in order to distinguish the NRGO samples from those that have been treated under inert atmospheres, the subsequent temperature of treatment as well as the employed gas will be written after the name, preceded by a hyphen, as it is recorded in Table 1.

A detailed list of sample names along with the employed treatment conditions is included in Table S1.

## 3. Results and Discussion

### 3.1. Synthesis of GO and N-Containing RGO (NRGO)

The morphology and size distribution of the GO precursor were determined by microscopy analyses. A SEM image of a GO specimen is shown in Figure S1. The dimensions of the oxidized sheets were measured from more than 200 images of GO. The flakes showed a quite homogeneous distribution with a mean area of 10.7 µm^2^ (lengths and widths in the range of 0.6–26.6 µm and 0.4–10.3 µm). The vast majority of GO sheets have areas between 1 µm^2^ and 25 µm^2^ (> 90%, inset Figure S1). Large size particles (>160 µm^2^) may correspond to material which oxidation was not complete during the synthesis.

Ammonolysis of GO was carried out in order to obtain NRGO functionalized with both aliphatic and structural moieties. GO was annealed at temperatures between 220 °C and 800 °C in the presence of a continuous flow of pure ammonia gas. In all the cases, the time of treatment and the flow of NH_3_ were kept constant at 1 h and 300 mL∙min^–1^ [29]. These conditions were selected to explore how the characteristics of the initial materials (N content and nature of the N-based groups) change with the annealing process. The composition and distribution of the N atoms in NRGO samples was determined by elemental analysis (EA) and X-ray photoelectron spectroscopy (XPS). EA confirmed the presence of nitrogen, with N contents of 9.7 wt.%, 9.6 wt.%, and 5.6 wt.% for NRGO samples prepared at 220 °C (NRGO220), 500 °C (NRGO500), and 800 °C (NRGO800), respectively (Table 2). XPS survey scan of GO reveals signals located at ca. 285 eV and ca. 530 eV, corresponding to C and O binding energies. Moreover, the appearance of a peak at ca. 400 eV, indicating the presence of N, was observed in the spectra of the ammonia treated samples. The appearance of a new signal when performing the deconvolution of the region corresponding to functionalized carbon (285.3 eV, high-resolution C1s spectra, Figure S2 and Table S2) suggests the modification of the GO surface by the introduction of N into the lattice. However, the level of reduction of the samples is directly related to the temperature of treatment, and the intensity of this region decreases due to the elimination of the O-bearing moieties. N1s XPS spectra of the NRGO samples (Figure S3) suggest the presence of three N-based groups, pyridinic N (ca. 398 eV), pyrrolic N (ca. 399 eV), and graphitic N (ca. 401 eV). In the case of the NRGO220 sample the presence of aliphatic N (amides and amines) cannot be discarded [21]. For further details on the characterization of the NRGO samples see supporting information.

### 3.2. Role of the Atmosphere Used in the Thermal Treatments of NRGO.

Initially, in order to analyze the role of the gas employed for the annealing treatments on the final content of nitrogen, NRGO500 was treated at 1050 °C under three non-oxidant atmospheres (N_2_, Ar, and Ar/H_2_). Since N_2_ has been used for the synthesis of nitride-based compounds, under plasma [8], and in order to determine any possible interaction of the oxygen-bearing functionalities with the flowing gas, a control sample was prepared by annealing GO under N_2_ (GO1050N_2_) following the same protocol applied to the N-doped samples. After this treatment, no significant changes in the nitrogen content are observed (0.3 wt.%, Table 2). Thus, the use of N_2_ gas is not expected to contribute towards the final nitrogen content in the NRGO500 post-treated samples. For the high temperature-treated NRGO samples (NRGO500-1050N_2_, NRGO500-1050Ar, NRGO500-1050Ar/H_2_, NRGO220-1050Ar, and NRGO800-1050Ar, see Table 2), some differences are observed in the final composition as a function of the employed gas. As expected, the samples showed a relative increase in the final content of carbon by the annealing treatment, as a consequence of the elimination of the aliphatic moieties present in their surface. From 81.3 wt.% in NRGO500 to 90–91.7 wt.% after the thermal treatments. In all the cases a decrease in the N content was observed, from 9.6 wt.% in NRGO500 to 4.9 wt.%, when this sample was treated under Ar and N_2_ (NRGO500-1050Ar and NRGO500-1050N_2_, respectively). This represents a 49% decrease in the nitrogen content of the sample. Even a more dramatic decrease (68.8% N) is observed when a reducing Ar/H_2_ atmosphere was employed (3 wt.% N, NRGO500-1050Ar/H_2_).

**Figure 1:**
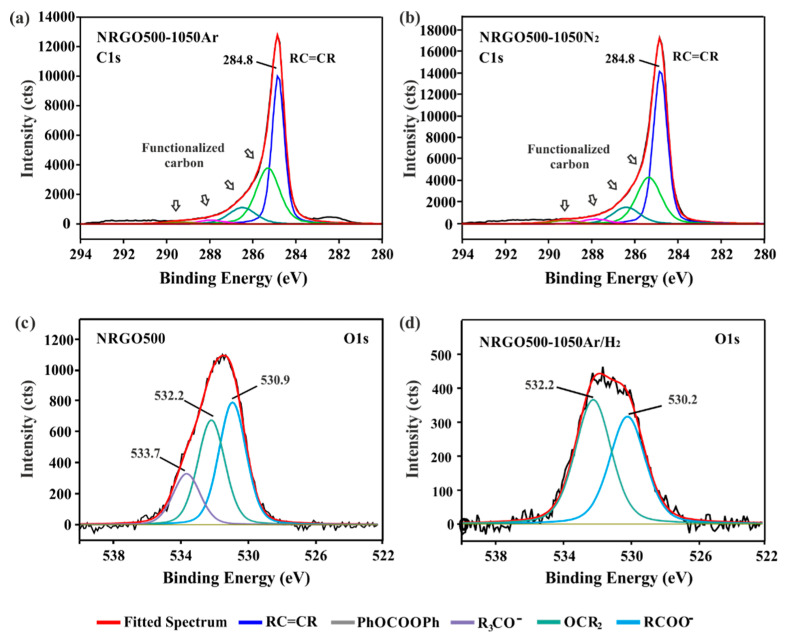
High-resolution C1s spectra of (**a**) NRGO500-1050Ar, (**b**) NRGO500-1050N_2_ and O1s spectra of (**c**) NRGO500, and (**d**) NRGO500-1050Ar/H_2_.

XPS is usually employed to discern between the functionalities present in graphene derivatives [8] because the binding energies of the core elements in the atoms are strongly dependent on their chemical environment. Small variations in the position of the peaks allow both the identification and the quantification of the chemical groups contained in the material. In the case of NRGO500-1050Ar and NRGO500-1050N_2_ no significant differences are appreciated in the distribution of both the sp^2^ band (RC=CR, 284.8 eV) and the signal corresponding to functionalized carbon atoms (which include C–N, C=O, C–O/C–O–C and COO^–^ groups, see Table S2) after the deconvolution of the high-resolution C1s spectra (Figure 1 a,b). However, the slight increase in the RC=CR signal when flowing N_2_ along the system might suggest the elimination of a larger fraction of O-based aliphatic groups from the sample.

On the other hand, the 1050 °C treatment under Ar/H_2_ of the NRGO500 sample induced more appreciable changes in the XPS spectrum of the material (NRGO500-1050Ar/H_2_). Figure 1 shows the deconvoluted high-resolution O1s spectra of the initial material (NRGO500, (c)) and the NRGO500-1050Ar/H_2_ sample (d). Drastic changes in the shape and even number of deconvoluted peaks are observed. A significant decrease in the concentration of the carboxylic and ketone groups is produced (50% and 30%, respectively). However, the most evident variation is the disappearance of the signal corresponding to the oxygen groups directly linked to the RGO lattice by sp^3^ bonds (R_3_CO–, Table 3).

Mikoushkin et al. have studied the hydrogenation of GO by thermal reduction (T > 750 °C) in H_2_ [41]. The elimination of the C–O groups may be induced either by the high temperature treatment (restoring the C=C conjugation) or by the introduction of hydrogen atoms (from the flowing gas) in the conjugated graphitic structure. Moreover, the hydrogen atoms can act as reducing agents of the oxidized aliphatic moieties. The hydrogenation process of the NRGO lattice may induce the formation of sp^3^ defects in the surroundings of the N-containing sites. This modification can alter the reactivity of the material favoring the elimination of nitrogen from the structure, which would explain the higher decrease in the nitrogen content of the NRGO500-1050Ar/H_2_ in comparison to the NRGO500 when treated in presence of Ar or N_2_.

**Figure 2:**
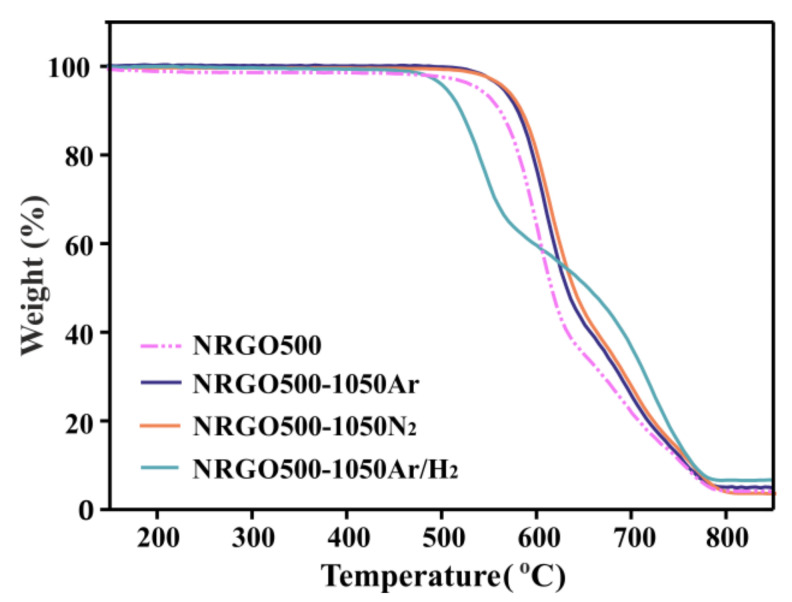
TGA of NRGO500 samples before (pink dashed line) and after (continuous lines) thermal annealing under N_2_, Ar, and Ar/H_2_ at 1050 °C. TGA were performed under flowing air at a heating rate of 10 °C·min^–1^.

TGA was next carried out to analyze the influence of the variations observed in the composition of the samples in their thermal stabilities (Figure 2, Table S3). For NRGO500-1050Ar/H_2_ (continuous green line), a 60 °C decrease in the onset of combustion (506 °C) was observed against the NRGO500 sample (pink dashed line). One could think that the reductive character of the flowing Ar/H_2_ should produce an enhanced thermal stability due to the highest elimination of oxygen-bearing moieties (2.9 at.% O, Table 3). The observed behavior confirms the determinant role of nitrogen in the thermal stability of RGO, because the combustion of the sample with the lowest N content (3 wt.%) is observed at the earliest temperature.

Focusing on the atomic composition of NRGO500-1050Ar and NRGO500-1050N_2_ slight differences can be observed. The samples contain similar amount of nitrogen, but the oxygen composition of the Ar-treated sample (8.5 at.% O) duplicates the amount determined in the sample treated under N_2_ (4.3 at.% O). A higher thermal stability would be expected for the NRGO500-1050N_2_ sample. Furthermore, if the same discernment is applied for all the materials, NRGO500 should be more stable than its counterpart treated under argon, due to its relatively lower oxygen content (6 at.% O). It is important to note that Ar, being an inert gas, does not introduce oxygen in the sample. Since Table 3 reflects the relative at.% content of C, N, and O, a decrease in the amount of nitrogen induces an apparent increase in the oxygen (and carbon) content of the sample. Analyzing the obtained results, the observed trend in the thermal stability of the samples cannot be explained a priori in terms of the total atomic composition of the samples. However, these results indicate that the thermal stability of the N-doped RGO (against oxidation by air) is mainly governed by the presence and nature of the N-based moieties and that in this case, the oxygen content plays a minor role.

**Figure 3:**
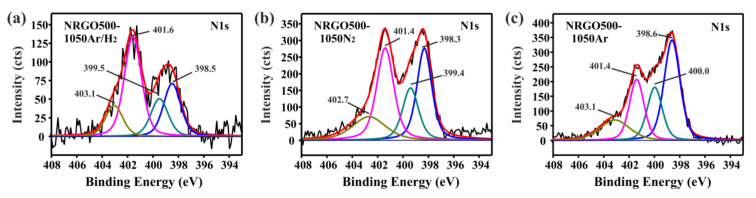
High-resolution N1s spectra of (**a**) NRGO500-1050Ar/H_2_, (**b**) NRGO500-1050N_2_, and (**c**) NRGO500-1050 Ar.

Considering that the functionalities present in NRGOs are responsible for the physicochemical properties of the materials (including their thermal stability), and that their presence is markedly affected by the temperature of the ammonolysis treatments, structural changes in the high temperature-treated samples are expected. Figure 3 shows the high resolution N1s XPS spectra of the NRGO500 after the 1050 °C post-synthesis treatments. Four signals corresponding to different types of N form the deconvoluted spectra. Apart from the frequently reported pyridinic, pyrrolic and graphitic N [42], an additional signal arising above 403 eV is observed (Table 3, Figure S3). The position of this peak corresponds to positive charged pyridinic N, usually stabilized by negative oxygen atoms (pyridine N-oxide) [43,44]. Beyond the appearance of a new signal, the most relevant change observed in the N1s spectra is the decrease of the pyrrolic and pyridinic N contents after the high temperature treatments. The thermal stability of the Ar and N_2_-treated samples is enhanced, despite the abrupt elimination of the nitrogen present in the five and six-membered rings. The observed increase in thermal stability could arise from the decrease of the pyrrolic species, which have been reported by Kundu et al. as the less stable of the structural nitrogens. The authors studied the thermal stabilities of N-containing carbon nanotubes [45], finding that ca. 49% of the pyrrolic N is already removed at 700 °C. The content of graphitic N remains almost invariable after the thermal treatment, which is in agreement with the high stability reported for these N-based moieties. The graphitic N is the most stable functionality when compared with pyridinic and pyrrolic N [45]. On the other hand, the six-membered structure of the pyridine N-oxide could also contribute to the stability of the sample. As discussed, NRGO500-1050Ar and NRGO500-1050N_2_ present differences in the amount of oxygen (8.5 at.% O and 4.3 at.% O, respectively). The similar thermal stabilities of the samples can be explained by the nitrogen present in the NRGO500-1050Ar (4.4 at.% N) and in NRGO500-1050N_2_ (3.7 at.% N). Moreover, the higher amount of six-membered rings that are more stable than five-membered cyclic moieties can also contribute to the stability of the Ar-treated samples. Finally, the significant decrease in the structural N-containing groups, and especially the elimination of a ca. 50% of the graphitic moieties might be responsible of the early combustion of the NRGO500-1050Ar/H_2_ sample. Pyridine N-oxide groups are probably generated by internal rearrangements or by the interaction of unstable nitrogen, contained in six-membered rings, with oxygen from the O-bearing functionalities remaining after the 500 °C ammonolysis treatment (6 at.% O).

### 3.3. Role of the Functionalities Present in the Starting Material

Taking into account that thermal treatments clearly induce both, the elimination of the less stable species and internal structural rearrangements, the nature of the initial functionalities could play a major role in the final structure of the samples. Both nitrogen-based aliphatic moieties and structural N doping are present in the NRGO220 lattice. After the ammonolysis at 220 °C, a high N content is achieved, whilst presenting a significant amount of O-bearing functionalities in the sample. Otherwise, NRGO800 presents the highest degree of reduction with all the N-based groups corresponding to structural species, namely pyridinic, pyrrolic and graphitic N. Due to the wide difference in the structural conformation of NRGO220 and NRGO800, these samples were annealed at temperatures ranged between 500 °C and 1050 °C under Ar. After the thermal annealing, both NRGO220 and NRGO800 present a relative increase in the amount of carbon, along with the removal of N (Table 2). The high decrease in the N content of NRGO220-1050Ar with respect to the former material NRGO220 (59.2%) could correspond to removal of the aliphatic moieties (amine or amide groups). The NRGO800 sample is strictly composed by N forming part of the RGO lattice. In this case the eliminated species correspond to pyridinic, pyrrolic, and graphitic N fractions.

**Figure 4:**
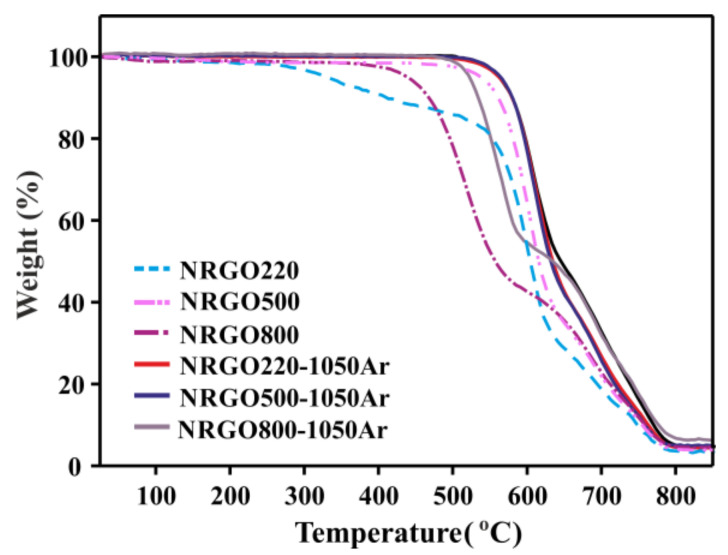
TGA of NRGO samples before (dashed lines) and after (continuous lines) thermal annealing under Ar at 1050 °C. TGA were performed under flowing air at a heating rate of 10 °C·min^–1^.

Figure 4 shows the TGA of NRGO220, NRGO500, and NRGO800 before and after annealing at 1050 °C under argon. The resulting samples are referred to as NRGO220-1050Ar, NRGO500-1050Ar, and NRGO800-1050Ar, respectively. All the high temperature treated samples show an enhanced thermal stability, compared to the former materials. However, the highest difference is observed for the NRGO220-1050Ar (red continuous line), due to the elimination of the aliphatic functionalities present in NRGO220 (blue dashed line), which is reflected by the disappearance of the continuous weight loss at ca. 220 °C. Furthermore, the onset of combustion of the NRGO220-1050Ar sample presents an increase, reaching a similar stability to the NRGO500-1050Ar sample (blue continuous line, Table S3). NRGO800-1050Ar (grey continuous line) presents the lowest onset of combustion (525 °C). When preparing NRGO, 800 °C is the most effective temperature to eliminate the aliphatic groups from GO but at this temperature the lowest introduction of nitrogen within the NRGO lattice is observed. The low concentration of N-based structural groups leads to a sample with relatively low thermal stability (5.5 at.%, NRGO800, Table 4), when compared with the NRGO500. The same trend is still observed after the high temperature treatments. Despite the onset of combustion of NRGO800 (471 °C) shows the highest increase (54 °C) after the 1050 °C treatment (525 °C, NRGO-1050Ar), the thermal stability of the resulting material under oxidizing conditions is still the lowest among this set of Ar-treated samples.

**Figure 5:**
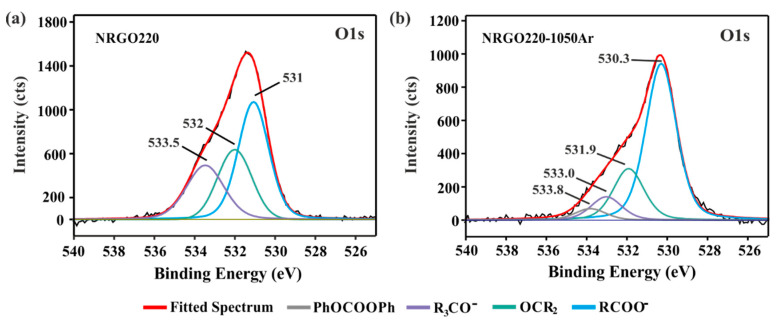
High resolution O1s spectra of (**a**) NRGO220 and (**b**) NRGO220-1050Ar.

Figure 5 shows the high resolution O1s XPS of NRGO220 (a) and NRGO220-1050Ar (b). A new peak (BE ca. 534 eV) is observed after annealing NRGO220 at 1050 °C. This signal can be assigned to aromatic derivatives (mainly PhOCOOPh groups). The formation of these groups could arise from the condensation reactions of phenolic functionalities still present after the initial treatment under ammonia.

The variation in the concentration of the functionalities can be also appreciated in the high resolution C1s data (Table 4). The 1050 °C treatments produce an increase in the C=C signal due to the elimination of nitrogen and oxygen-bearing moieties. Slight differences are observed in the N1s spectra. The presence of the pyridinic N-oxide groups (ca. 403 eV) is observed for both, NRGO220-1050Ar and NRGO800-1050Ar samples. Considering the numerical values obtained after the analysis of the data, similar amounts of nitrogen are observed in NRGO220-1050Ar and NRGO500-1050Ar (4.0 at.% and 4.4 at.% N, respectively). The atomic content of pyridinic, pyrrolic, graphitic N, and pyridine N-oxide groups is in agreement with the overlapping curves observed in the TGA. The high elimination of N-containing species in NRGO220-1050Ar with respect to NRGO220 (ca. 81% for the pyrrole/amine group) could be explained by the presence of N-aliphatic functionalities in NRGO220 (amides/amides). These groups are for instance not present in NRGO500 (Table 3). However, the signal corresponding to graphitic N results less affected by the thermal annealing. This confirms that this temperature (220 °C) is already useful to introduce structural functionalities when preparing NRGO, which indeed contribute to the high stability of the material against its oxidation by air. For the NRGO800-1050Ar the lower concentration of O-bearing functionalities in the starting material (NRGO800) produces the formation of a smaller amount of pyridine N-oxide groups (0.3 at.%).

### 3.4. Role of Temperature

**Figure 6:**
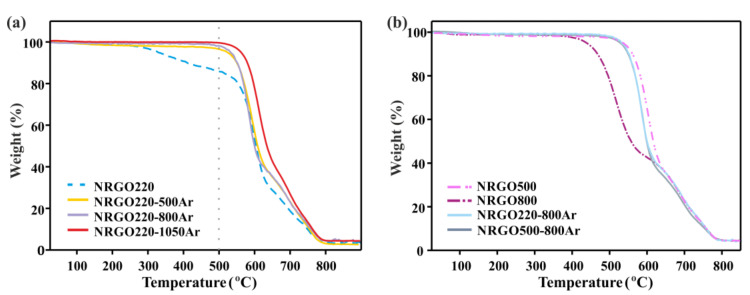
(**a**) Thermogravimetric analyses of NRGO220 sample before and after thermal treatment at 500 °C, 800 °C and 1050 °C under argon. (**b**) Thermal behavior against oxidation of NRGO220 and NRGO500 after thermal annealing at 800 °C under Ar (NRGO220-800Ar and NRGO500-800Ar). TGA of N-doped RGOs (NRGO500 and, NRGO800) are included for comparison. Analyses were performed under flowing air at a heating rate of 10 °C∙min^–1^.

In order to obtain a high elimination of the less thermally stable oxidized groups whilst minimizing the removal of the N-based functionalities, NRGO220 and NRGO500 were treated under Ar at lower temperatures. In all cases, the temperature employed for the treatment was higher than that employed for the preparation of the NRGO starting material. Figure 6a shows the TGA curves of NRGO220 before and after annealing under argon at 500 °C, 800 °C, and 1050 °C. After these treatments, the disappearance of the weight loss corresponding to the aliphatic fraction present in the NRGO220 sample (220–500 °C, blue dashed line) and a slight increase in the onset of complete combustion can be again appreciated (Table S3). These results are in agreement with the XPS data, where restoring of the conjugation is observed, as well as a decrease in the oxygen content when the temperature is increased (Table 4). Both, the 500 °C and the 800 °C post-annealed samples present similar thermal stabilities (NRGO220-500Ar and NRGO220-800Ar), and the highest onset of combustion is obtained for the sample treated at 1050 °C (NRGO220-1050Ar, 578 °C). A priori, a higher difference in the thermal behavior of the NRGO220-500Ar and NRGO220-800Ar samples would be expected due to the differences in their total N content (6.6 at.% and 5.4 at.%, respectively). The similar thermal stability might account for the level of reduction of the samples.

**Figure 7:**
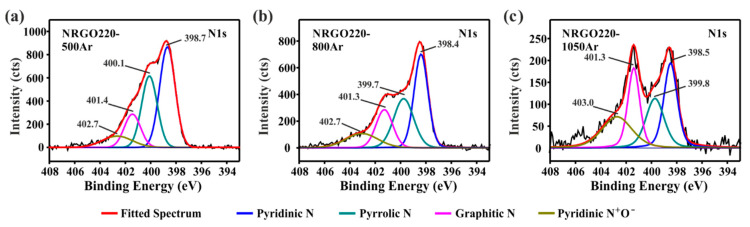
High-resolution N1s spectra of (**a**) NRGO220-500Ar, (**b**) NRGO220-800Ar, and (**c**) NRGO220-1050Ar.

Analysis of the high resolution N1s XPS of the NRGO220 Ar-treated samples reveals the presence of the pyridine-N-oxide species (BE ca. 403 eV, Figure 7a–c). The concentration of these groups is directly related with the temperature of treatment. Whereas graphitic-nitrogen groups maintain their concentration unchanged (ca. 1.0 at.%), a progressive decrease of the pyrrolic/amine signal is observed. The 500 °C treatment produced the removal of the aliphatic groups, but taking into account that the elimination of pyrrolic N is reported at mild conditions [45], the loss of these functionalities at 500 °C cannot be discarded. Higher temperatures of treatment (800 °C and 1050 °C) lead to the removal of structural N groups (pyridine and pyrrolic N).

It is worth nothing that the constant amount of the pyridinic/amide nitrogen after the 500 °C annealing reveals that internal rearrangements can occur when post-synthesis treatments are carried out. Thermal treatments produce the conversion of the aliphatic moieties into structural nitrogen (pyridinic N), which can be present in the form of neutral species or positive fractions stabilized by oxygen atoms. When NRGO220 is treated at 800 °C and 1050 °C the same trend is observed. However, the decrease in the 298 eV signal (pyridinic N) is attributed to the formation of positive charged groups by interaction with oxygen-containing moieties (0.7 at.% N) as well as the elimination of these groups from the NRGO lattice, which is favored at higher temperatures. Apparently, the presence of the pyridine N-oxide groups contributes to the enhancement of the thermal stability of the materials. The highest concentration of these functionalities is observed in NRGO220-1050Ar (0.9 at.% N) which, in turn, has the highest onset of combustion (578 °C, Table S3).

Treatment under argon at 800 °C produces samples with a high level of reduction (up to 51.1 at.% of sp^2^ C) and with a lower decrease in the N content compared to samples prepared by annealing at 1050 °C. In the case of NRGO220-800Ar and NRGO500-800Ar samples, the N content is similar to NRGO800 (5.4–5.8 at.%), but the post-annealed samples show the presence of pyridine N-oxide groups (Table 4). Again, these species contribute to the stabilization of the conjugated system, which would explain the higher thermal stability of the Ar-treated materials against NRGO800 (Figure 4).

The 500 °C and 800 °C argon-treated materials (NRGO220-500Ar and NRGO220-800Ar) show onsets of combustion close to NRGO500 (Table S3). Nevertheless, structural differences are clearly visible in the XPS analyses. NRGO500 has the highest contribution of stable six-membered-containing nitrogen rings. As discussed before, the stability of the sample is not governed by a single group. Considering the pyridinic N-oxide groups in NRGO220-500Ar, there are not large differences in the content of six-membered species between this sample and the NRGO500 (Table 4). The thermal stability of the samples does not show an increase when increasing the temperature from 500 °C until 800 °C under argon (Figure 6b). The treatment produces a slight decrease in the oxygen content (8.1–6.2 at.%) and a higher restoring of the conjugation (46.9–51.1 at.%, Table 4). Nevertheless, the decrease in the total content of nitrogen (from 6.6 at.% N in NRGO220-500Ar to 5.4 at.% N in NRGO220-800Ar) has a negative effect on the final onset of combustion of the materials.

When NRGO500 was treated at 800 °C under argon, the obtained material (NRGO500-800Ar) had a similar amount of nitrogen than NRGO220-800Ar, resulting in a similar onset of combustion. The formation of PhOCOOPh groups is favored in materials with higher content of oxygen, since they are not present in N-doped RGO treated under Ar at 800 °C (NRGO800). Interestingly, N-doping of GO at 800 °C and Ar treatment of already doped samples at 800 °C (NRGO220-800Ar, NRGO500-800Ar) lead to similar concentrations of nitrogen within the samples (5.5 at.% N, 5.4 at.% N, and 5.8 at.% N for NRGO800, NRGO220-800Ar, and NRGO500-800Ar, respectively). This indicates that the final content of nitrogen within the samples is mainly governed by the temperature of treatment.

**Figure 8:**
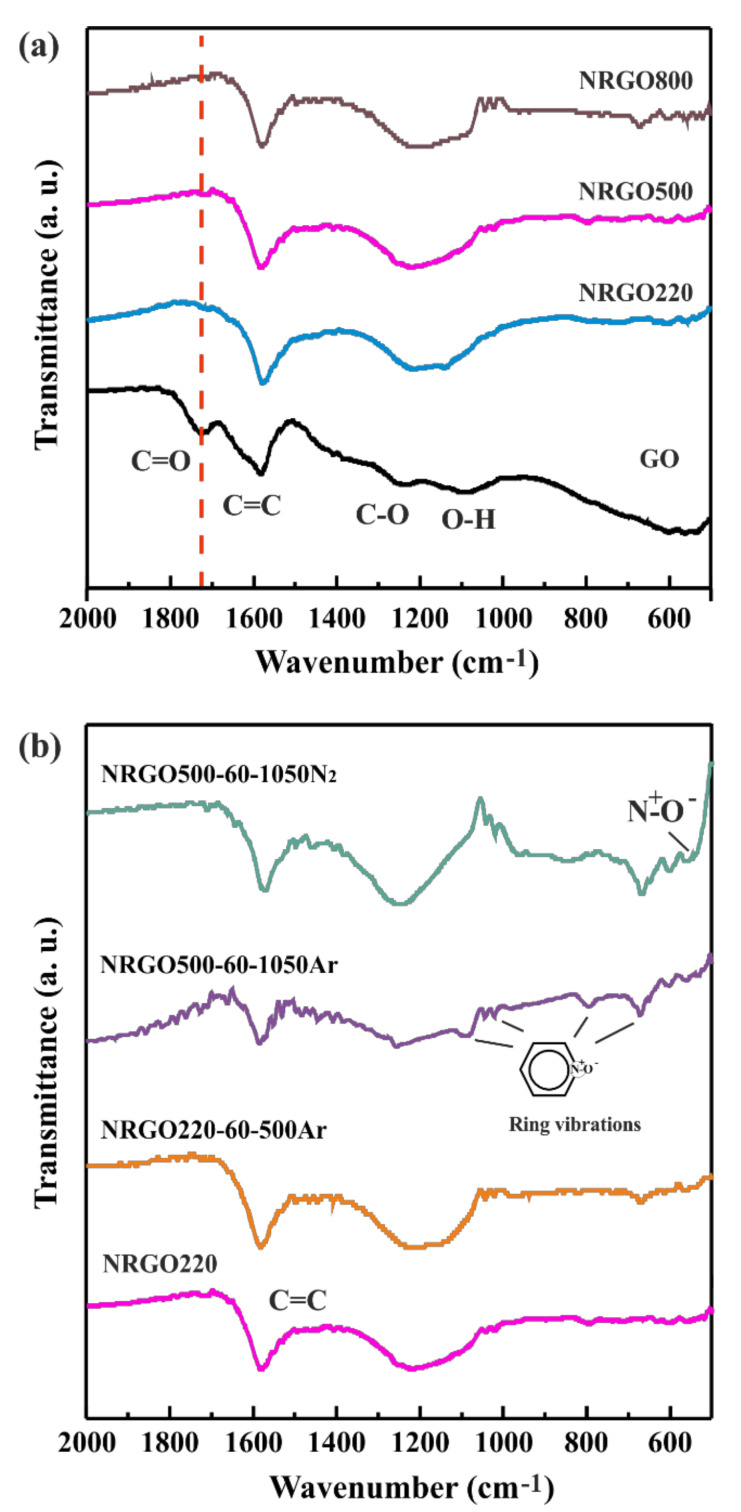
FT-IR of (**a**) GO and 220 °C, 500 °C, and 800 °C ammonia treated GO and (**b**) selected NRGO samples after high-temperature treatments under inert atmospheres.

To complete the study, FT-IR spectroscopy was performed to confirm the evolution of the N moieties present in GO and NRGO samples before (Figure 8 (a)) and after (b) high temperature treatments under inert atmospheres. As expected, GO (black line) shows both, signals corresponding to C=C stretching from the conjugated system (sp^2^ bonds, ν_C=C_ 1587 cm^–1^) and the characteristic stretching bands of oxidized aliphatic derivatives, namely, carboxylates (C=O, ν_C=O_ 1729 cm^−1^) and hydroxyl groups (C–O, ν_C-O_ 1238 cm^−1^). Finally, the signal resulting from bending vibrations of O–H species is also present (ν_O-H_ 1100 cm^−1^). After ammonia treatment (NRGO220, NRGO500, and NRGO800), the most significant variation in FT-IR spectra consist in the gradual elimination of the C=O signal. Partial removal of hydroxyl groups is also appreciated.

After annealing both NRGO220 and NRGO500 under argon at 500 °C and argon and N_2_ at 1050 °C, respectively, the appearance of a series of bands is observed in the range of 1100 cm^–1^ and 500 cm^–1^, with variable intensity. The new signals can be attributed to N^+^–O^-^ stretching (ν N^+^-O^-^ 552 cm^–1^), ring bending (669 cm^–1^) and ring stretching (1020 cm^–1^) vibrations of the pyridinic N-oxide fraction. The bands turn stronger when increasing the temperature of treatment, in line with results obtained after deconvolution of high-resolution N1s XPS spectra.

## 4. Conclusions

The distribution of N-based moieties present in N-containing RGO samples (NRGO) has been modified by high-temperature treatments under different atmospheres. The role of the concentration and nature of the N-species on the thermal properties of the final products has been evaluated. The thermal stability of NRGO samples is generally increased by annealing them under inert atmosphere in the 500–1050 °C range.

Ar/H_2_ treatments of NRGO produces the most abrupt decrease of N-containing structural moieties, namely graphitic N, pyrrolic N and pyridinic N; thus being responsible of the earlier combustion in air when compared with post-treated samples using N_2_ and Ar. The thermal annealing of NRGO samples induces inner rearrangements. Annealing the NRGO samples above 500 °C induces the elimination of the aliphatic fractions (amides and amines), which in some cases can be introduced into the conjugated lattice as structural groups (mainly pyridinic N). Additionally, N-containing six-membered rings can interact with oxygen atoms leading to the formation of pyridinic N-oxide species.

To conclude, thermal stability of N-doped GO is mainly governed by the nature of the N-groups, being favored by the presence of pyridine N-oxide moieties into the lattice. However, the restoration of the conjugated system via the elimination of O-bearing functionalities might also have a positive influence in the thermal response.

## Figures and Tables

**Table 1:** Designation chosen to distinguish the samples according to the corresponding synthesis, namely, ammonolysis of GO or post-synthesis thermal treatments.

Treatment (Precursor, Gas, T)	Name of the Sample
GO, NH_3_, 220 °C	NRGO220
NRGO220, Ar, 1050 °C	NRGO220-1050Ar

**Table 2:** Composition of C, N, and H (wt.%, determined by elemental analysis) present in GO and NRGO samples before and after annealing under N_2_, Ar, and Ar/H_2_.

Sample	C (wt.%)	N (wt.%)	H (wt.%)
NRGO220	71.5	9.7	1.3
NRGO500	81.3	9.6	0.9
NRGO800	85.7	5.6	0.7
GO1050N_2_	94.9	0.3	0.2
NRGO500-1050N_2_	90.0	4.9	0.2
NRGO500-1050Ar	91.0	4.9	0.3
NRGO500-1050Ar/H_2_	91.7	3.0	0.3
NRGO220-1050Ar	92.4	4.0	0.2
NRGO800-1050Ar	92.3	3.5	0.2

**Table 3:** Carbon, nitrogen, and oxygen atomic content (at.%, determined by XPS) present in NRGO500 after annealing this sample at 1050 °C under Ar (NRGO500-1050Ar), N_2_ (NRGO500-1050N_2_), and Ar/H_2_ (NRGO500-1050Ar/H_2_). In all cases the contribution of the specific functionalities is included.

Specie		Sample			
		NRGO500	NRGO500-1050Ar	NRGO500-1050N_2_	NRGO500-1050Ar/H_2_
C content (at.%)	RC=CR	43.1	42.3	49.7	60.4
	Functionalized carbon	42.4	44.8	42.3	35.4
	Total	85.5	87.1	92	95.8
N content (at.%)	Pyridinic N (C_5_N:)	3.8	1.8	1.1	0.3
	Pyrrolic N (C_4_N:)	3.4	0.9	0.7	0.2
	Graphitic N (C_4_N^+^)	1.3	1.0	1.3	0.6
	Pyridinic N^+^O^-^(C_4_N^+^-O^-^)	--	0.7	0.6	0.2
	Total	8.5	4.4	3.7	1.3
O content (at.%)	^-^O_2_CR	2.6	4.9	1.6	1.3
	OCR_2_	2.3	2.6	2.1	1.6
	^-^OCR_3_	1.1	1	0.6	--
	Total	6	8.5	4.3	2.9

**Table 4:** Carbon, oxygen, and nitrogen content (at.% determined by XPS) of NRGO (220–800 °C) before and after 1050 °C treatment under Ar. For comparison see data for NRGO500 in Table 3.

Specie		Sample						
		NRGO 220	NRGO 800	NRGO 220-500Ar	NRGO 220-800Ar	NRGO 220-1050Ar	NRGO 800-1050Ar	NRGO 500-800Ar
C content (at.%)	RC=CR	40.5	48.5	46.9	51.1	50.7	43.6	43.6
	Functionalized carbon	41.0	41.1	38.4	37.3	40.5	43.4	43.4
	**Total**	**81.5**	**89.6**	**85.3**	**88.4**	**91.2**	**87.0**	**87.0**
N content (at.%)	Pyridinic N	2.9 *	2.2	2.9	2.1	1.2	2.4	2.4
	Pyrrolic N	4.7 *	1.7	2.1	1.6	0.9	1.8	1.8
	Graphitic N	1.6	1.6	1.0	1.0	1.0	1.0	1.0
	Pyridinic N^+^-O^-^	--	--	0.6	0.7	0.9	0.6	0.6
	**Total**	**9.2**	**5.5**	**6.6**	**5.4**	**4.0**	**5.8**	**5.8**
O content (at.%)	^-^O_2_CR	4.3	1.5	4.0	3.2	3.1	4.1	4.1
	OCR_2_	2.6	2.7	3.8	2.0	1.0	2.1	2.1
	^-^OCR_3_	2.4	0.7	0.3	0.9	0.5	0.8	0.8
	PhOCOOPh	--	--	--	0.1	0.2	0.2	0.2
	**Total**	**9.3**	**4.9**	**8.1**	**6.2**	**4.8**	**7.2**	**7.2**

(*) These values could correspond to aliphatic functionalities (CONH_2_/CNH_2_) or to structural pyridinic (C_5_N:) and pyrrolic (C_4_N:) groups.

## Supplementary Materials

The following are available online at https://www.mdpi.com/2079-4991/10/8/1451/s1, Figure S1. SEM and statistical data, Figure S2. High resolution C1s XPS, Figure S3. High resolution N1s XPS, Figure S4. TGA, Table S1. Description of the synthesized samples, Table S2. Distribution of different functionalities observed after deconvolution of high-resolution C1s XPS signal and Table S2. Onsets of combustion.
