# Supplementary material for: Tuning the Nature of N-Based Groups From N-Containing Reduced Graphene Oxide: Enhanced Thermal Stability Using Post-Synthesis Treatments
Source: Nanomaterials (Basel). 2020 Jul 24;10(8):1451. doi: 10.3390/nano10081451 (PMC7466344; doi:10.3390/nano10081451)
Supplement: Supplementary file 1 [file nanomaterials-10-01451-s001.pdf]

nanomaterials

MDPI

Article

Tuning the Nature of N-Based Groups From N-Containing Reduced Graphene Oxide: Enhanced Thermal Stability Using Post-synthesis Treatments

Stefania Sandoval* and Gerard Tobias*

Institut de Ciència de Materials de Barcelona (ICMAB-CSIC), Campus de la UAB, 08193, Bellaterra (Barcelona), Spain

* Correspondence: ssandoval@icmab.es (SS), gerard.tobias@icmab.es (G.T.)

1. Description of the synthesized samples.

Table S1. Detailed sample names employed in the present study along the conditions of synthesis.

Sample	Synthesis conditions		
	**Precursor**	**Temperature (°C)**	**Atmosphere**
**NRGO220**	GO	220	NH_3
**NRGO500**	GO	500	NH_3
**NRGO800**	GO	800	NH_3
**GO1050N_2**	GO	1050	N_2
**NRGO220-500Ar**	NRGO220	500	Ar
**NRGO220-800Ar**	NRGO220	800	Ar
**NRGO220-1050Ar**	NRGO220	1050	Ar
**NRGO500-800Ar**	NRGO500	800	Ar
**NRGO500-1050N_2**	NRGO500	1050	N_2
**NRGO500-1050Ar**	NRGO500	1050	Ar
**NRGO500-1050Ar/H_2**	NRGO500	1050	Ar/H_2
**NRGO800-1050Ar**	NRGO800	1050	Ar

2. Characterization of GO and NRGO samples prepared by ammonolysis treatment.

2.1. Morphology of GO

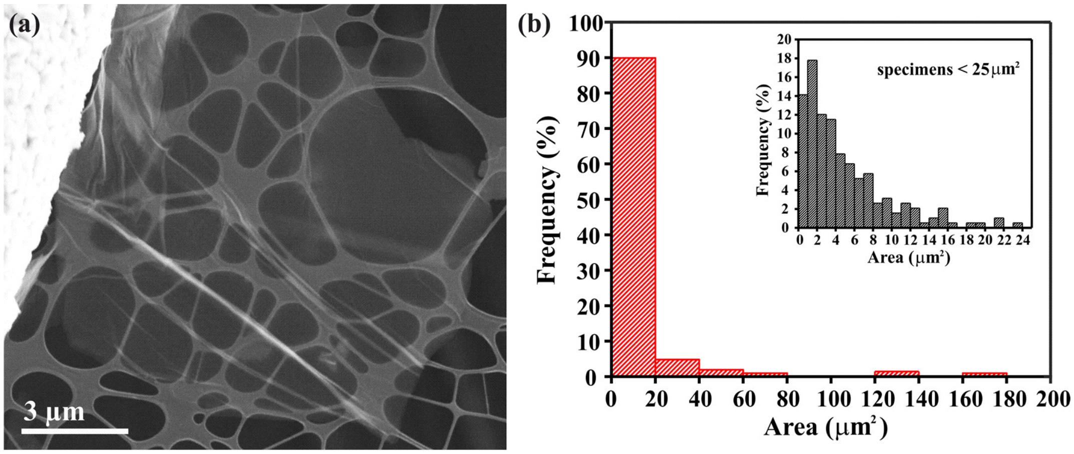

Figure S1. (a) SEM image of a GO specimen prepared from graphite flakes. (b) Histogram showing the GO size (area). *ca.* 200 GO specimens were measured from SEM. Size distribution of specimens with areas up to 25 μm² is included as inset.

2.2. Elemental composition and X-ray photoelectron spectroscopy

As expected GO shows a high content of O-bearing functionalities with a predominant presence of R–O- and R–C=O (59.6 % of the total area of the C1s signal). However, an abrupt decrease in the aliphatic moieties is observed, resulting from the elimination of the O-based aliphatic groups by the effect of the temperature treatment as well as the reducing character of the NH_3.

Table S2. Distribution of different functionalities observed after deconvolution of High-resolution C1s XPS signal (at. %). The position of each signal is included in brackets (binding energy, eV).

Sample	C content (at. %)					
	**RC=CR (284.8 eV)**	**C-N (285.3 eV)**	**C-O/C-O-C (286.3-286.5 eV)**	**C=O (287.6-287.9 eV)**	**-O-C=O (288.8-289.3 eV)**	**Functionalized carbon**
**NRGO220**	40.5	20.7	11.2	5.4	3.7	**41.0**
**NRGO500**	43.1	26.5	11.0	3.2	1.7	**42.4**
**NRGO800**	48.5	24.8	10.7	3.4	2.2	**41.1**
**NRGO500-1050Ar**	42.3	30.6	10.4	2.5	1.3	**44.8**
**NRGO500-1050N_2**	49.7	26.4	10.6	3.1	2.2	**42.3**
**NRGO500-1050Ar/H_2**	60.4	24.0	7.5	2.2	1.7	**35.4**

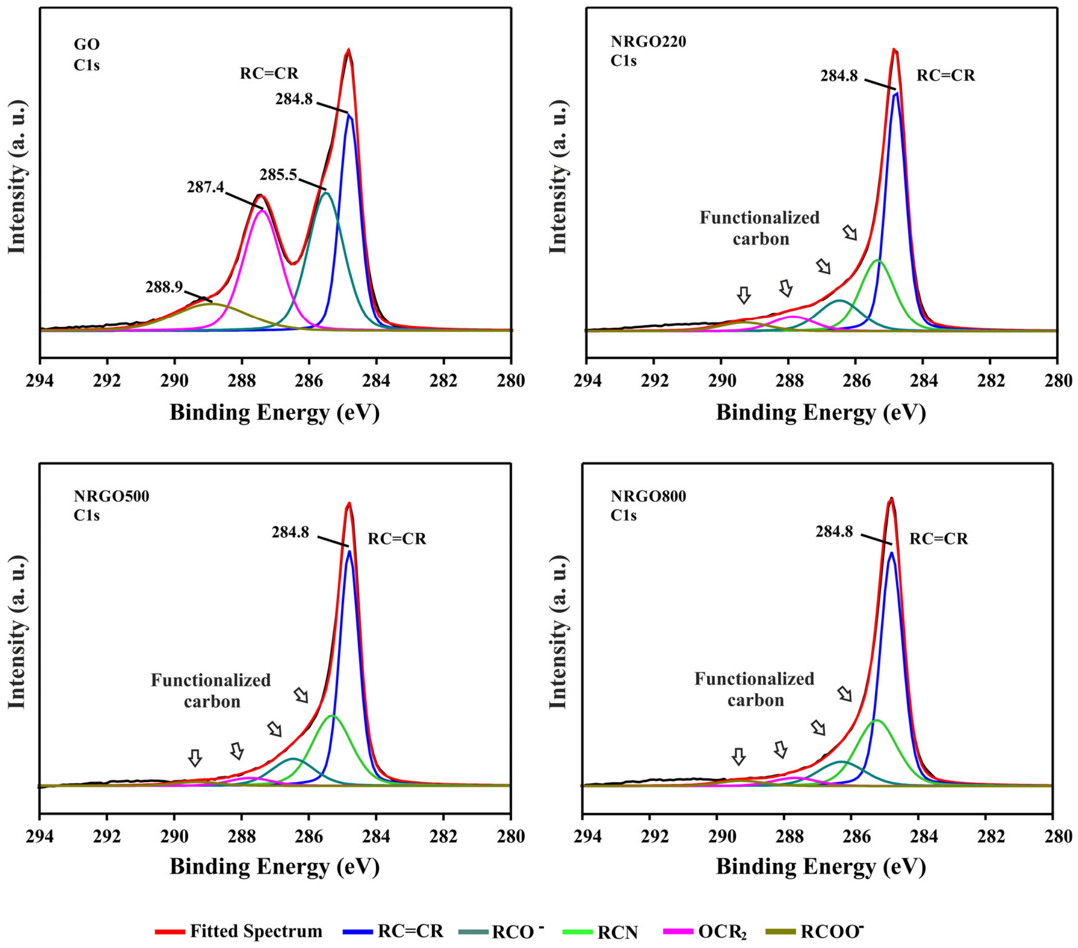

Figure S2. High-resolution C1s spectra of graphene oxide (GO) and graphene oxide after ammonolysis treatment at 220 ˚C (NRGO220), 500 ˚C (NRGO500) and 800 ˚C (NRGO800).

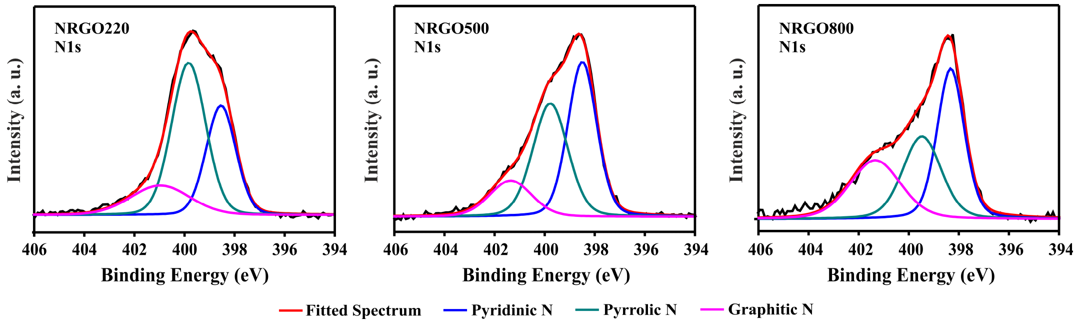

Figure S3. High resolution N1s spectra of graphene oxide after ammonolysis treatment at 220 ˚C (NRGO220), 500 °C (NRGO500) and 800 °C (NRGO800). The sample treated at 220 °C may also contain amine and amide groups, which overlap with the pyrrole and pyridine N signals.

3. Thermal stability of NRGO and high temperature treated RGO samples.

Figure S4 shows a comparison of TGA results of GO and the NRGO samples. Visual inspection of the TGA reveals an initial continuous weight loss under 200 °C for GO (usually attributed to atmospheric species, typically water), followed by a remarkable event starting at *ca.* 220 °C (25 wt.% loss) corresponding to the elimination of O-bearing moieties, mainly carboxylic and lactone groups. The total combustion of the sample starts at about 490 °C and mainly includes hydroxylic groups and carbon oxidation. The final weight loss arises from incompletely oxidized flakes, due to the large particle size of the starting material.

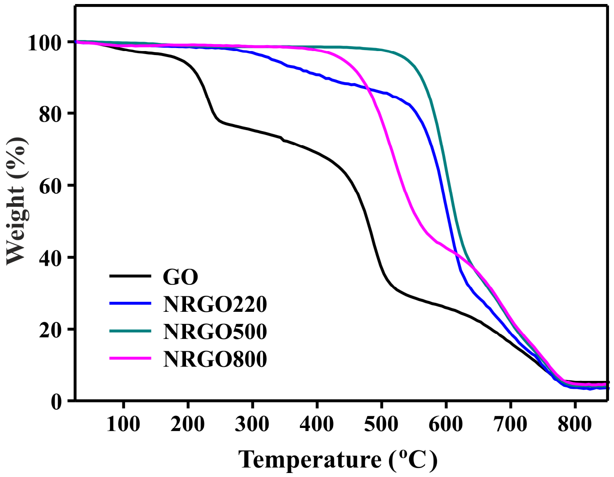

Figure S4. Thermogravimetric analyses of NRGO samples (220 °C, 500 °C and 800 °C). Thermal analysis of the starting material (GO) is included for comparison. Analyses were performed under flowing air at a heating rate of 10 °C·min^{-1}.

Table S3. Onset of combustion temperature in air of N-containing RGO samples before (NRGO220, NRGO500 and NRGO800) and after being thermally annealed (500-1050 °C) under different atmospheres (Ar, N_2 and Ar/H_2).

Sample	Onset of Combustion
NRGO220	549 °C (2nd weight loss)
NRGO500	566 °C
NRGO800	471 °C
NRGO220-500Ar	557 °C
NRGO220-800Ar	550 °C
NRGO220-1050Ar	578 °C
NRGO500-800Ar	559 °C
NRGO500-1050N_2	581 °C
NRGO500-1050Ar	578 °C
NRGO500-1050Ar/H_2	506 °C
NRGO800-1050Ar	525 °C

The disappearance of the first weight loss is produced when GO is treated under ammonia. The degree of reduction is directly related to the temperature employed for the treatments. The higher the N content of NRGO sample the higher the thermal stability against its oxidation by air (Table S3).
